# Bronchoscopic Drainage of a Persistent Lung Abscess Using CBCT‐Guided Aspiration Under Superimposed High‐Frequency Jet Ventilation

**DOI:** 10.1002/rcr2.70299

**Published:** 2025-08-03

**Authors:** Sammy Onyancha, Ahmad Sajad Soltani, Ramin Lonnes, Peter Hollaus, Waldemar Schreiner, Gernot Rohde

**Affiliations:** ^1^ Department of Pulmonology St. Elisabethen Krankenhaus Frankfurt Germany; ^2^ Department of Thoracic Surgery St. Elisabethen Krankenhaus Frankfurt Germany; ^3^ Department of Thoracic Surgery Goethe University Frankfurt, University Hospital Frankfurt Germany; ^4^ Department of Respiratory Medicine Universitätsklinikum Marburg Marburg Germany

**Keywords:** bronchoscopic drainage, cone‐beam CT, invasive pulmonary infection, jet ventilation, lung abscess, radial EBUS

## Abstract

Lung abscesses represent complex localised infections of the lung parenchyma. If they fail to resolve with conventional medical and surgical therapy, they pose a significant clinical challenge, particularly when the lesion is poorly accessible to percutaneous or open drainage. The evolution of advanced bronchoscopic techniques including cone‐beam computed tomography (CBCT) and superimposed high‐frequency jet ventilation (SHFJV) now allows for precise, minimally invasive interventions in such complex cases. We report a case of a persistent right upper lobe abscess due to invasive pulmonary infection that was successfully drained bronchoscopically. CBCT imaging enabled real‐time, three‐dimensional localisation and confirmation of needle placement within the abscess cavity. SHFJV, delivered through a jet converter system and endotracheal tube, stabilised the lung and minimised motion artefact during imaging and intervention. This case highlights the potential for bronchoscopic intervention in the multidisciplinary management of complex pulmonary infections. This procedure, performed entirely through flexible bronchoscopy, demonstrates how newer technical innovations enhance procedural accuracy, improve safety, and expand the therapeutic potential of interventional pulmonology beyond the traditional confines of rigid bronchoscopy.

## Graphical abstract

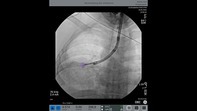

## 1 Introduction

Pulmonary abscesses are localised infections of the lung parenchyma that can occasionally prove refractory to conservative therapy with antibiotics. In such cases, drainage—typically through percutaneous—or surgical approaches is often necessary [1]. However, the anatomic location of the abscess, surrounding vascular structures, or adhesions may render traditional methods ineffective or unsafe.

Advances in therapeutic bronchoscopy have introduced new options for managing challenging cases. The use of cone‐beam computed tomography (CBCT) during bronchoscopy offers high‐resolution, intra‐procedural imaging with three‐dimensional reconstruction, allowing for precise localisation and verification of instrument placement within target lesions [2]. This facilitates safe navigation and enhances the confidence of interventionalists when working near critical structures or in previously inaccessible regions of the lung.

Superimposed high‐frequency jet ventilation (SHFJV) further complements these interventions by improving ventilation stability during bronchoscopy [3]. Although jet ventilation was traditionally limited to rigid bronchoscopic procedures [4, 5], it can now be administered through a jet converter system connected to an endotracheal tube or laryngeal mask, thus enabling its use during flexible bronchoscopy. This ventilation technique reduces respiratory motion and provides a more stable operative field during imaging acquisition and instrument manipulation, particularly critical when using CBCT, which requires motionless imaging windows for optimal resolution.

We describe a case where bronchoscopic needle aspiration under CBCT guidance and SHFJV was used to successfully drain a persistent lung abscess after failed surgical and percutaneous efforts.

## 2 Case Report

**FIGURE 1:**
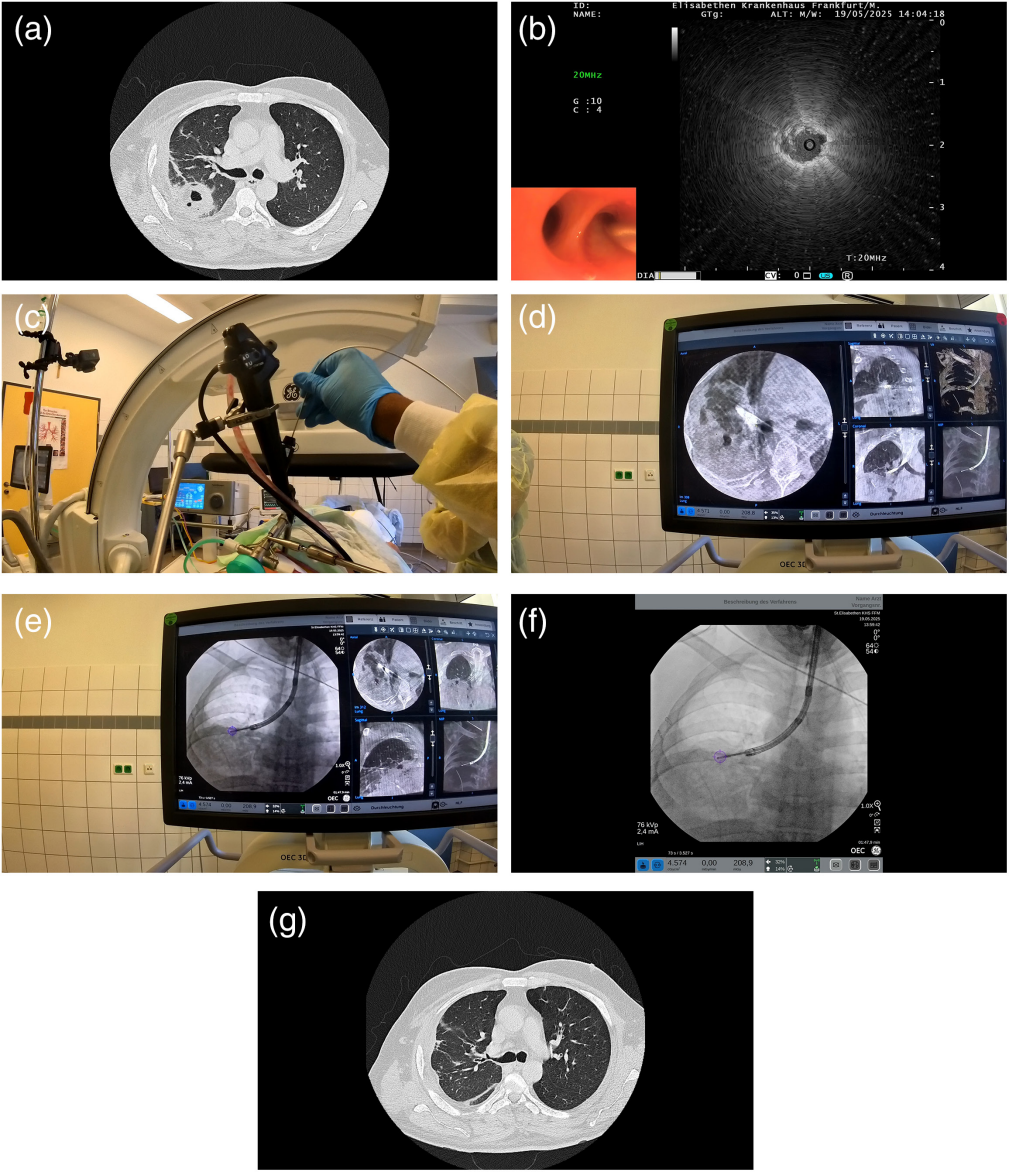
(a) Initial CT‐scan showing lung abscess in right upper lobe. (b) Positive rEBUS signal after locating upper lobe airway leading to lung abscess. (c) Aspiration needle being advanced into docked bronchoscope. (d) CBCT‐Imaging showing needle placement within lung abscess. (e, f) Aspiration of lung abscess under augmented fluoroscopy. (g) Follow‐up CT scan showing resolution of the upper lobe abscess.

A patient presented with a persistent right upper lobe lung abscess due to an invasive pulmonary infection (Figure 1a). Despite an extended course of broad‐spectrum antibiotics and multiple surgical interventions, including omentopexy, the abscess remained undrained. A chest tube placed for drainage proved ineffective.

Given the failure of conventional measures, a multidisciplinary decision involving pulmonology, thoracic surgery, and radiology was made to pursue a bronchoscopic approach. The decision to attempt bronchoscopic drainage was based on multidisciplinary consensus considering the central location of the abscess, proximity to accessible bronchial branches, and the failure of prior percutaneous and surgical drainage efforts. The risks of repeat surgery were considered prohibitive given the patient's recent operative history and comorbidities.

The patient was intubated with an endotracheal tube, and SHFJV was administered using a jet converter system to facilitate ventilation during the procedure. Radial endobronchial ultrasound (rEBUS) was employed to localise the abscess within the right upper lobe. Once the target was identified (Figure 1b), a Medi Globe aspiration needle was advanced through the bronchoscope to the site (Figure 1c). CBCT was then performed under a specialised ventilation protocol involving a prolonged inspiratory phase to minimise motion artefact. CBCT imaging confirmed accurate needle placement within the abscess cavity (Figure 1d). Multiple needle passes were performed under augmented fluoroscopic guidance to achieve drainage (Figure 1e,f) (Video 1). Approximately 35 mL of thick purulent material was aspirated in total. The total procedural time from intubation to completion was approximately 55 min.

**VIDEO 1:** Step by step—bronchoscopic drainage. Video content can be viewed at https://onlinelibrary.wiley.com/doi/10.1002/rcr2.70299.

A follow‐up CT scan performed 2 days post‐procedure demonstrated substantial radiological improvement with complete resolution of the previously persistent right upper lobe abscess (Figure 1g). The patient experienced no procedural complications and demonstrated continued clinical improvement. Following the procedure, the patient was continued on intravenous piperacillin‐tazobactam for 4 days, followed by an additional 3‐day course of oral amoxicillin‐clavulanate based on microbiological culture and clinical response.

Given the immediate decompression achieved, the absence of ongoing air leak or bronchopleural fistula, and the patient's improved clinical condition, prolonged drainage or bronchial valve placement was not deemed necessary.

## 3 Discussion

This case demonstrates the successful use of a bronchoscopic technique to drain a persistent lung abscess previously refractory to medical and surgical management. The combination of CBCT and SHFJV allowed for accurate and safe intervention through a flexible bronchoscope. SHFJV facilitated stable ventilation and minimised respiratory motion during needle placement, while CBCT provided real‐time imaging for confirming needle positioning, enhancing procedural safety and efficacy.

The decision to perform bronchoscopic drainage in this case, based on a combination of anatomical, clinical, and interventional considerations after multidisciplinary discussion, underscores the importance of multidisciplinary evaluation in selecting patients for novel interventions.

A key innovation in this case was the use of the jet converter system, which enabled the delivery of SHFJV through a conventional endotracheal tube rather than the rigid bronchoscope typically required for such ventilation strategies. This advancement signifies an important shift in interventional pulmonology, expanding the capability of flexible bronchoscopy beyond its traditional diagnostic boundaries.

Historically, interventions of this complexity, particularly those involving high‐frequency jet ventilation and deep parenchymal access, were confined to rigid bronchoscopy and therefore limited to centres with specific expertise and equipment. By enabling these complex procedures via flexible bronchoscopy, the use of devices like the jet converter reduces the technical and training barriers to entry. This offers access to a broader group of pulmonologists and centres, particularly those where rigid bronchoscopy training is limited. It also lowers the learning curve for advanced interventions, as mastery of rigid bronchoscopy is no longer a prerequisite.

This case highlights the evolution of therapeutic bronchoscopy made possible by innovations in imaging and ventilation. Bronchoscopic aspiration using this integrated approach represents a promising strategy for managing complex lung abscesses, especially when conventional approaches fail. It may serve as a minimally invasive alternative to surgical or percutaneous drainage in selected patients with refractory pulmonary abscesses. Further studies are warranted to explore the broader applicability and outcomes of this technique.

## Author Contributions

All the authors contributed to the manuscript. The first draft of the manuscript was written by Sammy Onyancha, and all the authors commented on previous versions of the manuscript. All the authors have read and approved the final manuscript.

## Ethics Statement

Written informed consent was obtained from the patient for publication of this case report and any accompanying images.

## Conflicts of Interest

The authors declare no conflicts of interest.

## Data Availability

The data that support the findings of this study are available from the corresponding author upon reasonable request.
